# Remineralizing amorphous calcium phosphate based composite resins: the influence of inert fillers on monomer conversion, polymerization shrinkage, and microhardness

**DOI:** 10.3325/cmj.2016.57.465

**Published:** 2016-10

**Authors:** Danijela Marović, Kristina Šariri, Nazif Demoli, Mira Ristić, Karl-Anton Hiller, Drago Škrtić, Martin Rosentritt, Gottfried Schmalz, Zrinka Tarle

**Affiliations:** 1 Department of Endodontics and Restorative Dentistry, School of Dental Medicine, University of Zagreb, Zagreb, Croatia; 2 Croatian Metrology Institute, Zagreb, Croatia; 3 Institute of Physics, Zagreb, Croatia; 4 Laboratory for Synthesis of New Materials, Division of Materials Chemistry, Ruđer Bošković Institute, Zagreb, Croatia; 5 Department of Operative Dentistry and Periodontology, University Hospital Regensburg, University of Regensburg, Regensburg, Germany; 6 Dr. Anthony Volpe Research Center, ADA Foundation, Gaithersburg, MD, USA; 7 Department of Prosthodontics, University Hospital Regensburg, University of Regensburg, Regensburg, Germany

## Abstract

**Aim:**

To determine if the addition of inert fillers to a bioactive dental restorative composite material affects its degree of conversion (DC), polymerization shrinkage (PS), and microhardness (HV).

**Methods:**

Three amorphous calcium phosphate (ACP)-based composite resins: without added fillers (0-ACP), with 10% of barium-glass fillers (Ba-ACP), and with 10% of silica fillers (Si-ACP), as well as commercial control (Ceram•X, Dentsply DeTrey) were tested in laboratory conditions. The amount of ACP (40%) and the composition of the resin mixture (based on ethoxylated bisphenol A dimethacrylate) was the same for all ACP materials. Fourier transform infrared spectroscopy was used to determine the DC (n = 40), 20 min and 72 h after polymerization. Linear PS and Vickers microhardness (n = 40) were also evaluated. The results were analyzed by paired samples *t* test, ANOVA, and one-way repeated measures ANOVA with Student-Newman-Keuls or Tukey’s post-hoc test (*P* = 0.05).

**Results:**

The addition of barium fillers significantly increased the DC (20 min) (75.84 ± 0.62%) in comparison to 0-ACP (73.92 ± 3.08%), but the addition of silica fillers lowered the DC (71.00 ± 0.57%). Ceram•X had the lowest DC (54.93 ± 1.00%) and linear PS (1.01 ± 0.24%) but the highest HV (20.73 ± 2.09). PS was significantly reduced (*P* < 0.010) in both Ba-ACP (1.13 ± 0.25%) and Si-ACP (1.17 ± 0.19%) compared to 0-ACP (1.43 ± 0.21%). HV was significantly higher in Si-ACP (12.82 ± 1.30) than in 0-ACP (10.54 ± 0.86) and Ba-ACP (10.75 ± 0.62) (*P* < 0.010).

**Conclusion:**

Incorporation of inert fillers to bioactive remineralizing composites enhanced their physical-mechanical performance in laboratory conditions. Both added fillers reduced the PS while maintaining high levels of the DC. Silica fillers additionally moderately improved the HV of ACP composites.

In the treatment of dental caries, dentistry has mostly been limited to removing the carious tissue and replacing it by a non-biological substitute. Even though the function and the esthetics of contemporary restorative materials are regarded to be fairly good, none of them is fulfilling all the requirements for an ideal replacement material (1) and their longevity is limited (2-4). Over the last few decades, increased efforts have been made to produce materials able to heal the carious process and remineralize the caries-affected tissue (5-8). Amorphous calcium phosphate (ACP) is the direct precursor to biological apatite in the biomineralization process.

ACP-based composite resins are bioactive dental materials that are able to actively participate in the natural process of carious tissue repair. They release calcium and phosphate ions in aqueous environments, such as the oral cavity, providing supersaturating concentrations sufficient to trigger apatite build-up. The remineralization process was demonstrated *in-vitro* (9-12), thus leading to the assumption that this class of materials could prevent secondary caries. This suggests their possible implementation as pit-and-fissure sealers, base liners, and direct restorative materials in non-load-bearing areas.

In most commercially available dental composite materials, fillers such as silanized glass or silica are used for their reinforcement so that the material could withstand masticatory stress. However, ACP is soft and porous and cannot fulfill this function in ACP composites. In order to use these materials in clinical practice as a restorative material, we have attempted to enhance their mechanical properties (13,14). By incorporating silanized inert fillers into a typical ACP formulation, flexural strength was improved, while optimal ion release needed for remineralization was retained.

Another issue that needs to be resolved is the high polymerization shrinkage (PS) of ACP composites. It is considered that fillings made with materials with high PS are prone to formation of large marginal gaps, which allow deeper penetration of bacteria, ultimately leading to pulp inflammation. Similar to other composite resins, ACP composites exhibit PS directly correlated with the high degree of conversion (DC) (15). The formation of macromolecular chain network from discrete monomer species involves the conversion of intermolecular distances into primary covalent bonds with much smaller interatomic distances. Volumetric PS of commercial composites varies from 1.5%-6% (16), while PS of ACP based composites is 4-8.5 vol.% (17) and it is attributed to their low filler load (40 wt.%). The incorporation of inert fillers is a possible means to decrease PS in ACP composites while improving some other physicochemical properties important for its implementation in clinical practice. For instance, material hardness is usually tested to predict its abrasion resistance if used as a restoration in functional areas (18,19).

However, this should not be done at the expense of the DC. A high DC is not only of utmost importance for ACP composite resins for providing better physical properties, but is also essential for the biocompatibility of such bioactive materials (20,21). The unpolymerized monomers can be released from the material under the conditions present in the oral cavity and cause toxic effects on pulpal and gingival cells (20). ACP composite resins usually exhibited high DC, equal or higher than other composite resins available on the market (55%-75%) (22). The DC of ACP composites can reach up to 90%, depending on the utilized resin system (23). Extrinsic factors such as light intensity and curing time taken aside, the DC of composite resins is strongly related to the composition of the resin matrix, the amount of filler, filler size, as well as the amount of silane adsorbed on the filler surface (24,25). Therefore, it is necessary to investigate if the added inert filler compromises the high DC of ACP resins.

This study is a continuation of the work of this group of authors that aims to create an ideal regenerative material for hard dental structures. So far, we have shown that the addition of certain inert fillers to ACP based composites improves their mechanical properties without reduction of their remineralizing potential (13,14). From a large pool of materials with different kinds of inert fillers and their amounts (13,14,21), the materials with the best results have been selected for further research. These materials are investigated in the current study, which tests if the same approach can be used to enhance the polymerization related properties of ACP composites, their PS, DC, and HV. The hypotheses were that the inert fillers: 1) had no influence on the DC, 2) reduced PS and 3) increased the HV of ACP composites.

## Materials and methods

### Materials

The synthesis of zirconia ACP (Zr-ACP) fillers followed the procedure by Skrtic et al (17). The experimental resin was made from ethoxylated bisphenol A dimethacrylate (EBPADMA; 62.8 wt%), triethylene glycol dimethacrylate (TEGDMA; 23.2 wt%), 2-hydroxyethyl methacrylate (HEMA; 10.4 wt%), methacryloxyethyl phthalate (2.6 wt%), camphorquinone (0.2 wt%), and ethyl-4-N,N-dimethylaminobenzoate (0.8 wt%). The monomers were acquired from Esstech (Essington, PA, USA) and camphorquinone and ethyl-4-N,N-dimethylaminobenzoate from Sigma Aldrich (Milwaukee, WI, USA).

The ACP fillers, silanized inert fillers (Table 1), and resin were mixed in lightproof containers in an asymmetrical centrifugal mixing device (Speed Mixer TM DAC 150 FVZ, Hauschild & Co KG, Hamm, Germany), and then pressed through three-roll machine (EXAKT 50, EXAKT, Norderstedt, Germany).

**Table 1:** Specifications of fillers added to the amorphous calcium phosphate (ACP) test materials

Fillers	Composition	Size (d50)*	Silanization (wt.%)*	Product name/ manufacturer
ACP	ACP 100%	5-8 μm	0	custom-made
Barium glass	Al_2_O_3_ 10.0% B_2_O_3_ 10.0% BaO 25.0% Fluorine, F 2.00% SiO_2_ 55.0%	0.77 μm	6	GM39923 Schott, Germany
Silica	SiO_2_≥99.8%	12 nm^†^	4-6	Aerosil DT, Evonik Degussa, Germany

*Data provided by the manufacturer.

†The primary particle size of silica fillers is 12 nm, but they do not exist in isolated form and they are agglomerated into clusters of approximately 12 μm (personal communication with the manufacturer).

The compositions of composite materials are shown in Table 2. Nanohybrid composite resin Ceram•X (Dentsply DeTrey, Konstanz, Germany; Mono+M5 shade; LOT 0910001050) was used as an external control material.

**Table 2:** The composition of the materials used in the study*

Material	Resin (wt.%)	Total filler (wt.%)		Total filler (vol.%)
0-ACP	60		40	27.5
Barium glass-ACP	50		50 (40% ACP and 10% barium glass)	35.0 (29.6% ACP and 5.5% barium glass)
Silica-ACP	50		50 (40% ACP and 10% silica)	36.5 (28.9% ACP and 7.6% silica)
Ceram•X	24		76	57

*ACP – amorphous calcium phosphate.

For the imaging of ACP composites, one sample (2 × 2 × 2 mm) from each material was made and photopolymerized for 40 s using a Bluephase C8 LED curing unit (Ivoclar Vivadent, Schaan, Liechtenstein; 1090 mW/cm^2^). The imaging was performed with a scanning electron microscope (SEM; Quanta FEG 400, FEI Company, Eindhoven, The Netherlands) in a low vacuum using a large-field detector and a back-scattered electron detector with an electric potential of 10 kV and 15 kV, at a working distance of approximately 10 mm.

### Degree of conversion

The composite samples (n = 10/group; d = 10 mm, h = 0.1 mm) were polymerized using a LED curing unit (Bluephase G2, Ivoclar Vivadent) for 30 s with irradiance 1150 mW/cm^2^. The uncured samples were pressed into KBr pellets (d = 1 cm) using spectroscopically pure KBr (Merck, Darmstadt, Germany). The DC was determined by a Fourier transform spectrometer (Mo. 2000, Perkin Elmer, Beaconsfield, Bucks, UK).

The spectra of un-polymerized and polymerized composite specimens were recorded in a transmission mode at room temperature, corrected by subtracting the background and then converted into the absorbance mode. 20 scans per sample were measured at a resolution of 4 cm^-1^. Two measurements were performed per sample: 20 min and 72 h after the polymerization of samples.

The peak ratios were calculated according to Rueggeberg’s baseline method (26). The DC (%) was calculated from the equivalent aliphatic (1638 cm^-1^)/aromatic (1610 cm^-1^) molar ratios of cured (C) and uncured (U) samples according to the following formula:

DC = (1 – C/U) × 100 (%)

### Polymerization shrinkage

Linear polymerization contraction was evaluated using laser interferometry (27). A coherent light beam from a helium-neon laser (Spectra Physics, Santa Clara, CA, USA; λ = 632.8 nm, maximum power 25 mW) was first expanded by a lens and then divided into object and reference beams. The reference beam was steered directly to the charge-coupled device (CCD) sensor (Conrad Electronics, Hirschau; Germany; 500 × 582 pixels, 50 Hz), while the object beam was first directed onto a mirror positioned on the upper sample surface and then steered to the CCD sensor. Both beams were recombined creating an interference pattern whose fringes are stationary when the sample mirror is not moving. Any change in the sample thickness causes mirror movement and consequently fringe shifts. The change in sample thickness, as well as polymerization contraction ratio was determined by numerical evaluation of the fringe shifts applying the Fast Fourier Transform (FFT) method.

The composite material was placed between two PET films to obtain a 0.85 mm thickness. The unpolymerized sample (n = 10/group) was placed on a glass plate, with half of the sample covered with the object mirror. The curing unit was positioned under the glass plate, and the sample material was polymerized for 30 s through the glass plate. The dimensional changes in the sample thickness were recorded during photopolymerization and continued for another 30 s after the end of polymerization. The phase information was obtained from the interference of the two laser beams. Based on phase information, the displacement of the object mirror was numerically calculated using FFT method. It corresponded to the change in the sample thickness and the linear polymerization contraction of the material.

### Microhardness

The samples made for the measurement of linear polymerization contraction were also used for measuring Vickers microhardness. The specimens were kept in a dry and dark place at room temperature for one week before HV testing. The Leitz Miniload 2 Microhardness Tester (Leitz, Oberkochen, Germany) was used for the HV measurement with loads of 5 and 10 g. Each of the 10 polymerized samples per material was subjected to three measurements for each load. The data from different loads and locations of measurements were pooled and expressed as means ± standard deviation (SD) for each material.

### Statistical analysis

The DC data (10 samples/4 materials/2 time points) were expressed as means ±SD. The normality of the distribution was tested using Kolmogorov-Smirnov test. DC values obtained 20 min and 72 h after polymerization were analyzed by paired samples *t* test, one-way analysis of variance (ANOVA), and one-way repeated measures ANOVA with Student-Newman-Keuls post-hoc test. For estimation of effect size partial η^2^ was used. The PS data (10 samples/4 materials) and HV data (10 samples/4 materials) were expressed as means±SD and the differences between the groups were analyzed using ANOVA and Tukey’s post-hoc test. All analyses were performed in SPSS 10.0 software (SPSS Inc., Chicago, IL, USA) with the significance level set at *P* < 0.05.

## Results

**Figure 1:**
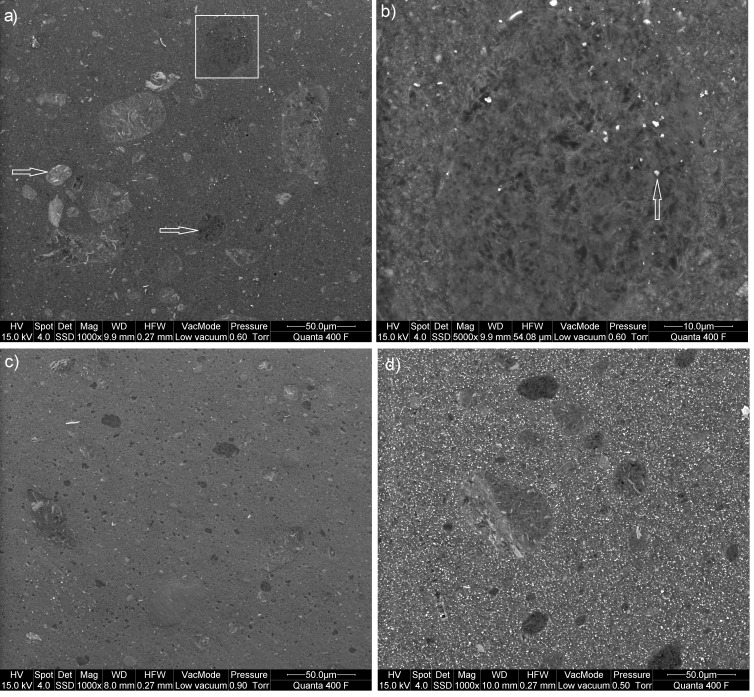
(**A**) Micromorphology of the 0-amorphous calcium phosphate (ACP) material. Dark and light ACP particles (arrows). (**B**) The area inside the square is magnified, showing the porous structure of a large ACP particle. The white dots (arrow) are residual Al_2_O_3_ particles from polishing. (**C**) Silica-ACP. Silica fillers are not visible. (**D**) Barium-glass-ACP with its highly packed structure.

The micromorphology of the ACP composite resins is represented in Figure 1. The 0-ACP contains dark and light particles (Figure 1A). Dark particles show the porous structure of ACP (Figure 1B), while the lighter (more electron-dense) particles show a linear structure. In the Si-ACP material (Figure 1C), the low-atomic weight silicon cannot be seen, therefore, the distribution of the added Si particles cannot be evaluated as in the Ba-ACP (Figure 1D). The Ba-ACP sample shows the homogenous structure and densely packed fillers.

**Figure 2:**
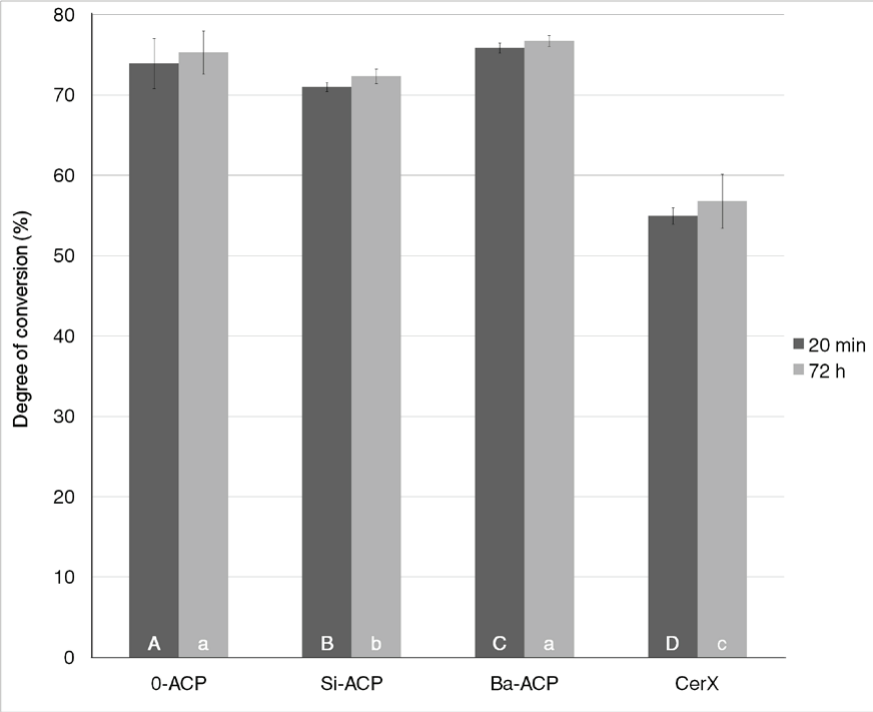
The degree of conversion (mean ± standard deviation). Same letters indicate no significant differences between samples; the uppercase letters refer to values after 20 min and lowercase letters after 72 h.

There was a significant difference between the two different time points for each tested material (*P* < 0.001, Student-Newman-Keuls post-hoc test), except for Ceram•X. The highest polymerized material after 20 min was Ba-ACP. At that time point, the addition of barium fillers significantly increased the DC (75.84 ± 0.62%) in comparison to 0-ACP (73.92 ± 3.08%), but the addition of silica fillers lowered the DC (71.00 ± 0.57%). Ceram•X had the lowest DC (54.93 ± 1.00%). After 72 h, there was no difference between Ba-ACP (76.73 ± 0.68) and 0-ACP (75.28 ± 2.69) due to large post-cure DC increase in 0-ACP. Si-ACP had lower DC (72.32 ± 0.91) than other ACP composites, but Ceram•X was the most poorly polymerized material (56.76 ± 3.32) at 72 h (Figure 2).

**Figure 3:**
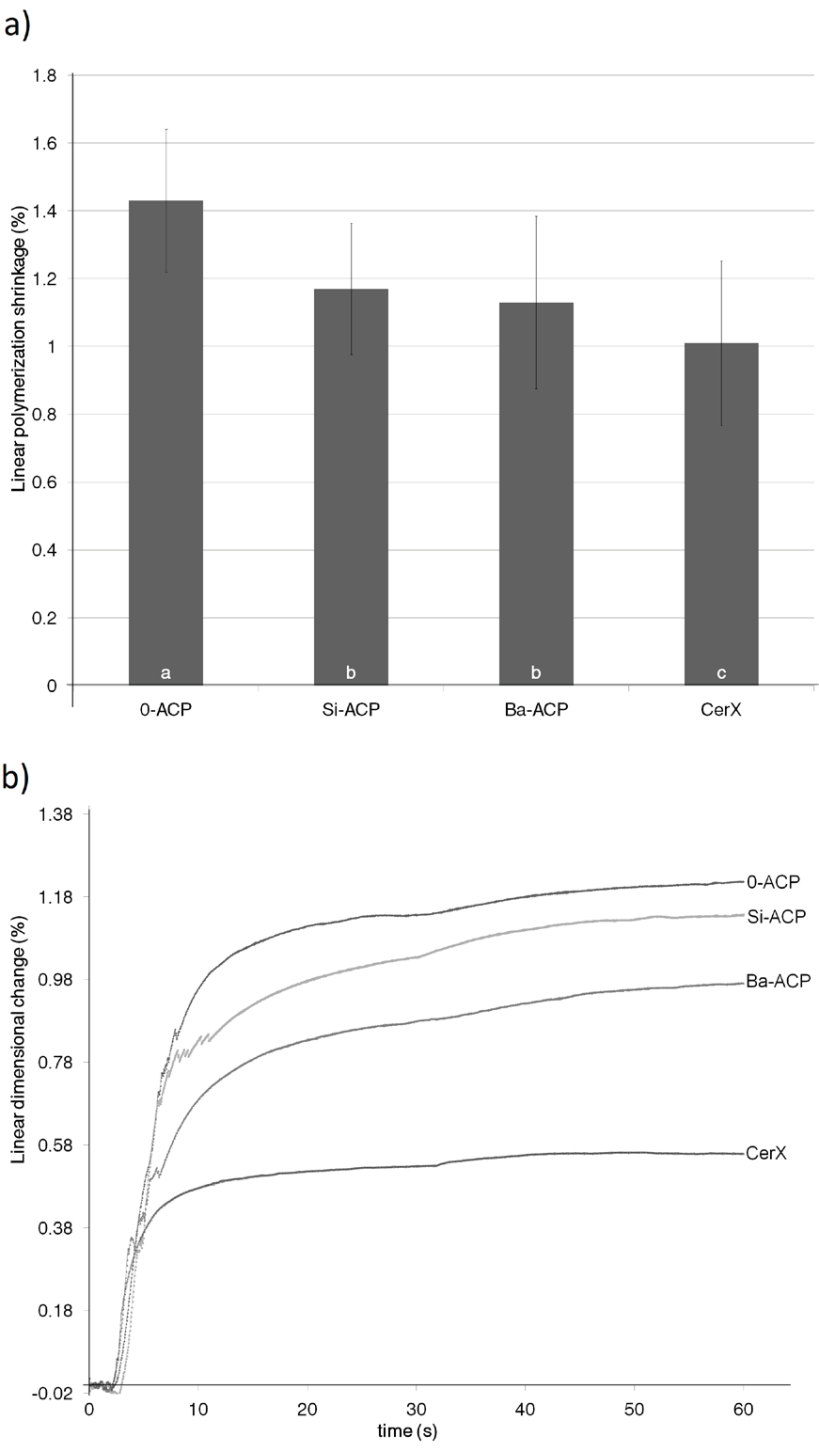
Linear dimensional changes of tested materials during polymerization and 30 s after: (**A**) Linear polymerization shrinkage (mean ± standard deviation). Different letters indicate a significant difference between the materials. (**B**) The examples of the experimentally obtained polymerization shrinkage curves for each tested material as a function of time.

All materials demonstrated initial expansion and consecutive contraction, which continued after the end of the polymerization (Figure 3). Commercial control Ceram•X had the lowest PS (1.01 ± 0.24%; *P* < 0.01, Tukey’s post-hoc test). 0-ACP with no inert fillers had a significantly higher PS (1.43 ± 0.21%) than Ba-ACP (1.13 ± 0.25%) or Si-ACP (1.17 ± 0.19%) (*P* < 0.010), with no significant difference between the latter two.

**Figure 4:**
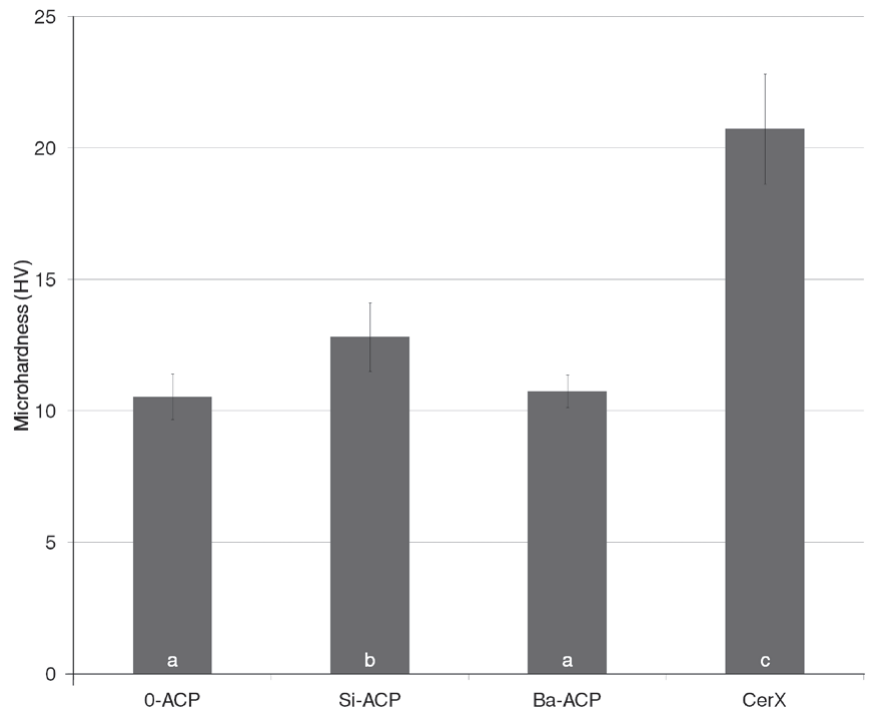
The microhardness of the tested materials (mean ± standard deviation). Different letters indicate a significant difference in microhardness between test materials (*P* < 0.010 for all equations except for 0-ACP and Ba-ACP).

A significant difference in HV was found between Si-ACP and the other two ACP containing materials (*P* < 0.010, Tukey’s post-hoc test), as well as between Ceram•X and all ACP containing materials (Figure 4). Ceram•X exhibited the highest HV (20.73 ± 2.09). Si-ACP had significantly higher HV (12.82 ± 1.30) than 0-ACP (10.54 ± 0.86) and Ba-ACP (10.75 ± 0.62) (*P* < 0.010).

## Discussion

The inclusion of inert fillers significantly reduced PS, but their effect on DC and HV of bioactive ACP composite resins depended on the type of added filler. Thus, the working hypothesis was partially rejected.

Both ACP composites with inert fillers reduced PS compared to 0-ACP, with no significant difference between them. The benefit of increased filler volume is related to the smaller amount of resin monomers that can polymerize (28). Therefore, similar volumetric percentage of fillers in Si-ACP and Ba-ACP reduced the PS compared to 0-ACP, but did not lead to significant difference between them. This is also evident for Ceram•X, which has a double amount of fillers compared to 0-ACP and 30%-35% more fillers than Ba-ACP and Si-ACP. This has also contributed to a low DC value due to increased light dissipation. Relatively low PS values for the control material can be attributed to low DC values as well.

Unlike dilatometry, which shows only the final values of a change in the volume of a sample, laser interferometry records the dimensional changes real-time (29). This property enabled the visualization of the initial expansion at the start of the polymerization process in all tested materials, which was followed by the contraction. The initial expansion can be explained by the transient thermal expansion at the start of polymerization. It is well known that composite polymerization is an exothermic reaction. Also, the heat transfer from the high-intensity curing unit, such as the one used in this study, contributes to the temperature rise within the composite sample. This initial expansion is later masked by a much higher polymerization contraction. Our results confirm the findings of Lau et al (30), who also used real-time measurement of polymerization shrinkage kinetics by digital image correlation.

Polymerization kinetics in biphasic filler systems is more complex than in composite resins with a single type of fillers. This is especially the case in this particular system with dominating ACP fillers. Even though ACP is zirconia-hybridized to enhance its interaction to the resin, there is no silanization and their adhesion to resin is minimal (31). Inert filler particles added in relatively small amounts to the composition of ACP composites are similar in degree of silanization, but they differ in size and surface area. Thus, there is less restriction in monomer mobility during polymerization and relatively high DC is obtained. This is also the most probable reason for a significant post-cure polymerization increase in the DC noted for all ACP materials in this study. Unsilanized ACP particles presumably allowed certain monomer mobility even after the curing period had finished. A similar effect was observed in composites filled with particles coated with non-functional silane (32).

Surprisingly, Ba-ACP achieved higher DC values 20 min after the initiation of photopolymerization than the control 0-ACP. However, the high post-cure increase in the DC for 0-ACP evened out the differences after 72 h. Disparate refractive indices (RIs) of resin and fillers make materials appear opalescent and increase light scattering within the material, whereas similar RIs contribute to translucency and improve light conduction (33). Presumably, the RIs of the resin matrix and barium-glass fillers were well matched. EBPADMA has a RI of 1.53, whereas HEMA and TEGDMA have RIs of 1.45 and 1.46, respectively, which makes the end RI of the resin matrix probably lower than 1.53, and to some extent, similar to the RI of barium-glass fillers (1.52). This observation is consistent with our previous work, where light transmittance was measured in very similar materials to 0-ACP and Ba-ACP and no difference was found between them (34).

Also, a lower DC of Si-ACP than of 0-ACP could be explained by the increased light scattering of silica fillers, as demonstrated previously (34). If well dispersed in the composite, nanoparticles do not contribute to scattering (35). However, this is often not the case, since they tend to agglomerate (33). Despite the efforts to achieve homogenous filler particle distribution in all composite pastes, filler clusters in Si-ACP might have been formed due to agglomeration tendency of both ACP and silica, but also due to the relatively high amount of silica fillers. Low-viscosity materials tend to achieve higher DC than high-viscosity materials (36). This was especially notable in highly viscous Si-ACP, probably because of the relatively high amount of silanized silica nanoparticles with extremely high surface areas (160 m^2^/g vs 13 m^2^/g for barium-glass fillers). In a viscous medium, the macroradicals mobility is restricted, and the diffusion-limited termination occurs, thus leading to a lower DC. Such a composition might be the reason of its poor predictability regarding the DC. In our previous study, Si-ACP achieved higher DC than Ba-ACP after 72 h (21). Different curing protocols (higher irradiance and a shorter illumination time in the present study) might have influenced this discrepancy. However, it should be noted that the DC decrease of approximately 3% is clinically negligible and that this sacrifice is minor in terms of benefits of reduced shrinkage and better mechanical properties.

The material with the lowest DC, Ceram•X, demonstrated the highest HV and the materials with the highest DC showed the lowest HV. The HV values obtained for ACP materials were half as high as those for Ceram•X, which is attributable to its high glass-filler content (37). Si-ACP was the only ACP material that achieved a significant increase in HV compared to ACP control. Even though this value is small, it shows a trend of increase. Ba-ACP did not demonstrate increased HV, regardless of the highest DC. The effect of higher DC and higher total filler volume in this material was probably diminished by the fact that this material contained more ACP than the 0-ACP (29.6% vs 27.5%). A previous study demonstrated that the addition of ACP reduced certain mechanical properties in comparison to pure resin without any fillers (38). A similar amount of ACP was also present in the Si-ACP (28.9%), but it was more heavily loaded with inert fillers (7.6% silica fillers in Si-ACP vs 5.5% of glass fillers in Ba-ACP). Silica fillers with high surface area are able to form a stronger bond to the resin and, in turn, achieve higher HV values (39). This increase, however, is still not sufficient for clinical application of these materials, and further investigations with trimodal filler mixtures are needed.

There are several limitations of this study. First, a homogeneous distribution of larger amount of silica fillers is very hard to achieve outside factory due to their agglomeration tendency. This makes their clinical application unpredictable in this composition. Also, certain laboratory techniques that use spot measurements, such as HV, show very different values even in the same sample. More importantly, this observation is noted in the commercial control material, which suggests that other methods might be more suitable for the evaluation of material’s mechanical properties. The flexural strength and modulus, used in our previous studies (13,14) might be more appropriate. Second, it would be beneficial to simultaneously measure the DC and PS on the same sample, or at least to gather the DC and PS data on different samples of the same material at the same time. This would make the conclusions on the polymerization properties more accurate. However, our current laboratory setup makes this impossible at the moment.

This study confirmed that the inclusion of silanized inert fillers in the original ACP formulation had a beneficial influence on the reduction of PS of ACP-based composite resins. Barium-glass microfillers increased DC and reduced PS, whereas silica nanofilers reduced PS and additionally improved the HV. However, the achieved improvements are still insufficient for the clinical application of the materials, and further work needs to be directed to incorporation of larger amounts of inert fillers and optimization of the active/inert filler ratio.
